# Competitive traits of coral symbionts may alter the structure and function of the microbiome

**DOI:** 10.1038/s41396-020-0697-0

**Published:** 2020-06-09

**Authors:** Shelby E. McIlroy, Jane C. Y. Wong, David M. Baker

**Affiliations:** 1 The Swire Institute of Marine Science, The University of Hong Kong, Hong Kong, Hong Kong, PRC; 2 School of Biological Sciences, The University of Hong Kong, Hong Kong, Hong Kong, PRC

**Keywords:** Microbial ecology, Stable isotope analysis

## Abstract

In the face of global warming and unprecedented coral bleaching, a new avenue of research is focused on relatively rare algal symbionts and their ability to confer thermal tolerance to their host by association. Yet, thermal tolerance is just one of many physiological attributes inherent to the diversity of symbiodinians, a result of millions of years of competition and niche partitioning. Here, we revealed that competition among cocultured symbiodinians alters nutrient assimilation and compound production with species-specific responses. For *Cladocopium goreaui*, a species ubiquitous within stable coral associations, temperature stress increased sensitivity to competition eliciting a shift toward investment in cell replication, i.e., putative niche exploitation. Meanwhile, competition led *Durusdinium trenchii*, a thermally tolerant “background” symbiodinian, to divert resources from immediate growth to storage. As such, competition may be driving the dominance of *C. goreaui* outside of temperature stress, the destabilization of symbioses under thermal stress, the repopulation of coral tissues by *D. trenchii* following bleaching, and ultimately undermine the efficacy of symbiont turnover as an adaptive mechanism.

## Introduction

In marine phytoplankton, interspecific variability in metabolic responses to light, temperature, and nutrients are important drivers of competition and community assembly [1–3]. Indeed, constraints on growth by limiting nutrients such as nitrogen and phosphorus can have profound implications for species dominance in the surface ocean, and downstream consequences for ecosystem functioning. For example, Burson et al. [2] showed that variation in nitrogen and phosphorus loads could induce single or co-limitation of resources (both nutrients and light), which altered the community composition of phytoplankton. Nitrogen in particular is broadly limiting to life on Earth as it is the fundamental building block for amino and nuclei acids—both critical for cell division and growth. It is therefore curious that despite decades of accumulated knowledge on the impact of nutrients on coral reef ecosystems, there have been few studies to examine the role of nutrient competition on *in hospite* communities of dinoflagellates.

Endosymbiotic dinoflagellates from the genetically diverse lineage *Symbiodiniaceae* [4] supply the majority of a coral’s energy (carbon; [5]) and growth (nitrogen; [6, 7]) resources through photosynthesis and tight recycling of the host’s metabolic waste. Indeed, emerging evidence suggests that host modulation of nitrogen to the symbionts keeps them in a nitrogen-limited state [8]—a condition which is well-known to slow cell division and induce the accumulation of carbohydrates and lipid compounds with relatively high C:N ratios in marine phytoplankton [9, 10]. While coral species/individuals tend to associate with one or a few predictable, dominant symbiodinians, these associations can vary with depth (light availability) and temperature. *In hospite* distributions of symbiodinian species along light gradients optimize interspecific variability in photosynthetic performance [11, 12], with niche partitioning along micro-scales [13, 14] and macro-scales [15]. Meanwhile, some  species persist at background levels while providing no discernible function ([16]; but see [17]). It is these background types, generally within the genus *Durusdinium*, which transition in and out of dominance following disturbance [18]. Interestingly, these opportunists are often associated with thermal tolerance [19]. While variation in functional traits of symbionts is clearly an important determinant of holobiont physiology [20], it can also drive the outcomes of ecological interactions among those microbes, and the subsequent efficacy of microbial turnover as an acclimatory mechanism. This raises an important question: is competition among symbionts limiting the proliferation of these thermally tolerant species in an ever-warming world, and in particular, how do the different symbiodinians compete?

One of the reasons that ecological interactions among microbes are still poorly understood is the difficulty in isolating the function of specific microbes within a complex microbiome [21]. To understand competition between *Symbiodiniaceae* species, we traced the assimilation of isotopically enriched carbon and nitrogen in cultures of two common co-associates of *Acropora* spp. (*Cladocopium goreaui* and *Durusdinium trenchii*). Where *C. goreaui* and *D. trenchii* were grown together within the same flask (i.e., mixed species cultures), we used a combination of species-specific fluorescent in situ hybridization (FISH) labeling and flow cytometric sorting to separate cells of each species (Flow) with subsequent stable isotope analysis (SIA). We hypothesized that the carbon and nitrogen assimilation and resulting changes in their stoichiometry  (C:N) would be distinct for each species in ways that could underpin their ecological distributions, and that these metabolic traits could be modified in response to the presence of a competitor. To assess this, the FISH-Flow-SIA methodology was applied to a fully crossed experimental design with temperature (26 and 32 °C) and interspecific competition (present and absent) as factors.

## Materials and methods

### Culture isotope enrichment pulse

Cultures of *C. goreaui* and *D. trenchii* were acquired from the Symbiont Culture Facility at the Australian Institute of Marine Science (see Table S1 for details). Cultures were subsequently maintained in batch culture at 26 °C and 120 µmol photons m^−2^ s^−1^ with monthly transfers into 0.22 μm filtered artificial seawater (Instant Ocean Spectrum Brands) supplemented with f/2 media (Gillard 1975). For isotopically enriched pulses, f/2 media was amended with Na^13^CO_3_ (98% ^13^C, Sigma; [H^13^CO_3_^−^] **≈** 1.18 mM) and Na^15^NO_3_ (98% ^15^N, Sigma; [NO_3_^−^] **=** 3.88 μM). To each flask we added 100 mL of enriched media and ~10,000,000 total cells per flask among the following treatments: *C. goreaui* monoculture (*n* **=** 8), *D. trenchii* monoculture (*n* **=** 8), *C. goreaui* + *D. trenchii* (0.5:0.5) coculture (*n* **=** 16). The increase in coculture replicates was in anticipation of potential data loss in splitting the sample for species-specific labeling, which can result in too low of sample mass for SIA analyses. Furthermore, two of the coculture replicates were analyzed in bulk (without FISH-Flow) for methods development (ESM Appendix 1; Fig. S3). After 4 h of incubation under 200 µmol photons m^−2^ s^−1^ at 26 or 32 °C, samples were processed in batches under low light and in a random order to limit any bias of additional nutrient assimilation during sample processing. We transferred cells to 50 mL falcon tubes, centrifuged at 5000 × *g* for 5 min, washed twice with deionized water, and fixed with 5X SET Buffer (3.75 mol l^−1^ NaCl, 25 mmol l^−1^ Na_2_EDTA, 0.5 mol l^−1^ Tris, pH 7.8). Samples were then stored at −80 °C until further analyses. Experimental light level was chosen to avoid light limitation and was confirmed with rapid light curves on individual cultures to be below potential stress levels (ESM Fig. S5).

### FISH protocol

Samples were treated with a modified protocol from [22]. Immediately preceding hybridization, samples were thawed and washed sequentially in 5X SET with IGEPAL at 0.4 and 0.1% vol/vol. Samples were split among four tubes to decrease cell concentrations and increase probe efficiency. For hybridization, samples were incubated overnight at 45 °C in hybridization buffer (5X SET, 0.1% vol/vol IGEPAL, 10% vol/vol formamide) with 100 pmol of a probe. *C. goreaui* monoculture and *D. trenchii* monoculture were hybridized with SymC and SymD [22], respectively, while *C. goreaui* + *D. trenchii* coculture treatments were split and hybridized with SymC and SymD separately. The following morning, samples were centrifuged and washed with warm 1X SET for 5 min, vortexed and resuspended in 500 ul of 1X SET at room temperature, and subjected to cytometric analysis within 1–5 h.

### Flow cytometry and sorting

All samples were analyzed and sorted on a BD Influx (BD Biosciences, San Jose, CA). ACCUDROP beads (BD Biosciences) were used to set drop delay at the start of each session. A gating hierarchy was used to set the conditions for sorting samples. First a gate was used to screen for cells of the appropriate shape and size (FSC-A vs. SSC-A; Fig. S1a), then two gates were used to remove doublets (FSC-W vs. FSC-H and SSC-W vs. SSC-H; Fig. S1b, c). Gating of probe- positive cells within a treatment was dependent on their position within the 582 nm vs. SSC-A (Fig. S1d, e); the gated cells were sorted using two-way yield mode.

### Stable isotope analysis

The sorted cells were rinsed twice with DI water and harvested by centrifugation (4000 × *g*, 5 min, 4 °C). The resulting pellets were transferred to pre-tarred tin capsule and oven dried at 60 °C overnight. To ensure that N masses were above the detection limit for reliable δ^15^N values, 0.18 mg of nitrogen carrier (=42.6 µM of NaNO_3_) was added to each sample before analysis [23]. Samples were combusted in an elemental analyzer (Eurovector EA3028) coupled to a stable isotope ratio mass spectrometer (Nu Instruments Perspective) in continuous flow mode. Internal standards (acetanilide; Indiana University, IN, USA) were measured to normalized sample δ^15^N and δ^13^C values. Mean precision for δ^15^N and δ^13^C were 0.25‰ and 0.07‰, respectively. Isotopic enrichment was corrected to account for dilution by the carrier. Due to uncertainty in developing novel methodologies, data occasionally fell outside of the range of detection because either not enough sample mass was obtained for ^15^N readings, or the ^13^C signal was oversaturated due to user error in helium dilution settings. The resulting sample sizes are reported in Table 1.

**Table 1:** Samples sizes for carbon and nitrogen analyses.

Temperature (°C)	Competition	Species	*n* for AP^13^C	*n* for AP ^15^N
26	Absent	*C.g*.	8	8
26	Present	*C.g*.	14	14
32	Absent	*C.g*.	2	8
32	Present	*C.g*.	12	8
26	Absent	*D.t*.	7	8
26	Present	*D.t*.	13	13
32	Absent	*D.t*.	2	8
32	Present	*D.t*.	12	12

Experimental replicates for the experiment were *Cladocopium goreaui* (*C.g*.) monoculture (*n* = 8), *Durusdinium trenchii* (*D.t*.) monoculture (*n* = 8), *C. goreaui* + *D. trenchii* coculture (*n* = 14). However, due to uncertainty in developing novel methodologies, data occasionally fell outside of the range of detection because either not enough sample mass was obtained for ^15^N readings, or the ^13^C signal was oversaturated due to user error in helium dilution settings.

Detailed methodological development and validations of FISH-Flow-SIA are provided in ESM Appendix 1 and Figs. S1–S4.

### Statistics

All analyses were run in base R version 3.5.1 (R Core Team 2018). Analysis of variance was used to determine the response of variables to fixed factors as outlined below. For each test, assumptions of ANOVA were tested. Hedge’s *g* was used to report effect sizes to account for variation in sample sizes.

#### Effects of methodology on stable isotope readings

A one-way ANOVA was used to compare atom percent (AP) values of ^13^C and ^15^N of samples collected and analyzed directly after experiment and those subjected to FISH labeling and flow cytometry (see Appendix S1).

#### Functional differences

Data from monocultures of *C. goreaui* and *D. trenchii* were included in a two-factor ANOVA with species and temperature as fixed factors and response variable of either AP ^13^C or AP ^15^N.

#### Competition

To understand the effect of competition on AP ^13^C and AP ^15^N, we subset the data for each species and ran a two-factor ANOVA on each dataset using temperature and competition (presence/absence) as fixed factors.

#### Stoichiometry of response

The ^13^C to ^15^N ratio was calculated for each sample. After subsetting the data for each species, the effects of temperature and competition for each species were determined with ANOVA.

## Results

### Assumptions of statistical tests

When testing the effect of temperature and competition on AP ^15^N for *C. goreaui*, we found a significant violation of homogeneity of variance (Levene’s test *p* = 0.002). Three outliers spread across treatments were identified. When these were excluded, the remaining dataset satisfied the assumption of homogeneity of variance (Levene’s test *p* = 0.39). Subsequent ANOVAs on this dataset with and without these outliers included  resulted in the same statistical significance outcomes. Therefore, we retained the full dataset for statistical reporting and data plots. Despite variation in sample size among treatments (Table 1) all other statistical comparisons met the assumptions of the ANOVA.

### Functional variability

**Fig. 1:**
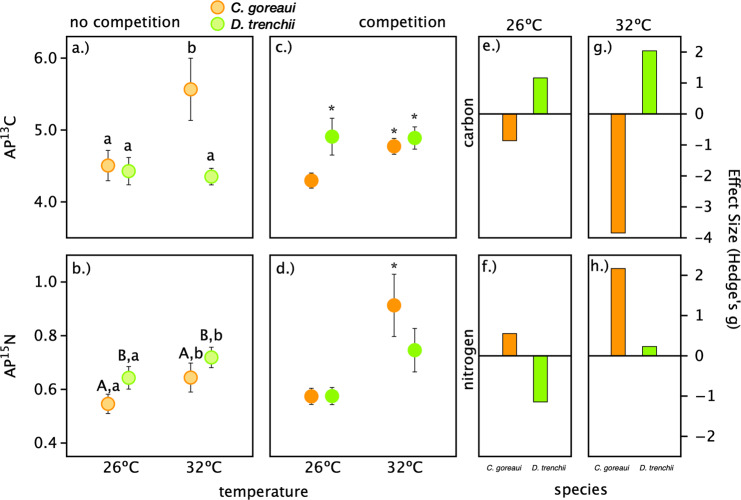
Functional differences and the response to competition. Atom percent (AP) of carbon (^13^C) and nitrogen (^15^N) in *Symbiodiniaceae* cultures *Cladocopium goreaui* (orange) and *Durusdinium trenchii* (green) incubated in tracer enriched media for 4 h at 26 or 32 °C in the absence (**a**, **b**) or presence (**c**, **d**) of interspecific competition. Means are shown with 95% confidence intervals as error bars. Small letters in (**a**) and (**b**) indicate significant differences between species and temperature based on two-factor ANOVA (species × temperature) and Tukey’s HSD post hoc analyses (*p* < 0.05). Asterisks in (**c**) and (**d**) indicate significant effects of competition relative to data collected from monocultures of that species (**a**, **b**; two-factor ANOVA, competition × temperature). The effect size (Hedge’s *g*) of competition on each species at each temperature is shown in (**e**–**h**).

Two-factor ANOVA on AP ^13^C data revealed a significant effect of temperature (*F*_(15,1)_ = 6.68, *p* = 0.02), and a significant temperature by species interaction (*F*_(15,1)_ = 8.6, *p* = 0.01; Fig. 1a). At the 26 °C baseline, carbon assimilation for both *C. goreaui* and *D. trenchii* was similar (with mean ± SD of AP ^13^C 4.51 ± 0.30 and 4.43 ± 0.26, respectively). At 32 °C, AP ^13^C in *C. goreaui* increased by 24% (AP^13^C = 5.57 ± 0.31), which was significantly higher than AP ^13^C in comparison with each of the other baseline treatments (Tukey’s HSD *p* < 0.02). For AP ^15^N, ANOVA revealed a significant temperature (*F*_(28,1)_ = 22.4, *p* < 0.001) and species (*F*_(28,1)_ = 21.9, *p* < 0.001) effect with no interaction (*p* = 0.53; Fig. 1b). At 26 °C, *D. trenchii* had a higher rate of nitrogen assimilation (AP ^15^N 0.64 ± 0.06) relative to *C. goreaui* (AP ^15^N 0.55 ± 0.04). The relative increase in nitrogen assimilation at 32 °C for *C. goreaui* and *D. trenchii* was 18% and 12%, respectively (Fig. 1b).

### Interspecific competition

For *C. goreaui*, there was a significant effect of competition (*F*_(1,32)_ = 5.6, *p* = 0.02), temperature (*F*_(1,32)_ = 53.7, *p* < 0.001), and a competition × temperature interaction (*F*_(1,32)_ = 8.2, *p* = 0.007) on AP ^15^C (Fig. 1c). Post hoc comparisons revealed that carbon assimilation at 32 °C was significantly decreased in response to the presence of a competitor (Tukey’s HSD *p* < 0.001; Hedge’s *g* = −3.84; Fig. 1g). For AP ^15^N, there was a significant effect of competition (*F*_(1,34)_ = 12.4, *p* = 0.001), temperature (*F*_(1,34)_ = 63.0, *p* < 0.001), and a competition × temperature interaction (*F*_(1,34)_ = 16.5, *p* < 0.001; Fig. 1d). Post hoc comparisons showed that at 32 °C AP ^15^N in *C. goreaui* was significantly higher when competition was present (Tukey’s HSD *p* < 0.001; Hedge’s *g* = 2.17; Fig. 1h). For *D. trenchii*, AP ^13^C was significantly affected by competition (*F*_(1,30)_ = 10.5, *p* = 0.002), but not temperature (*p* = 0.83), nor a significant interaction term (*p* = 0.87; Fig. 1c). Contrastingly, AP ^15^N was significantly affected by temperature (*F*_(1,37)_ = 21.6, *p* < 0.001), but not competition (*p* = 0.43), nor their interaction (*p* = 0.11; Fig. 1d).

### Stoichiometry of the response

**Fig. 2:**
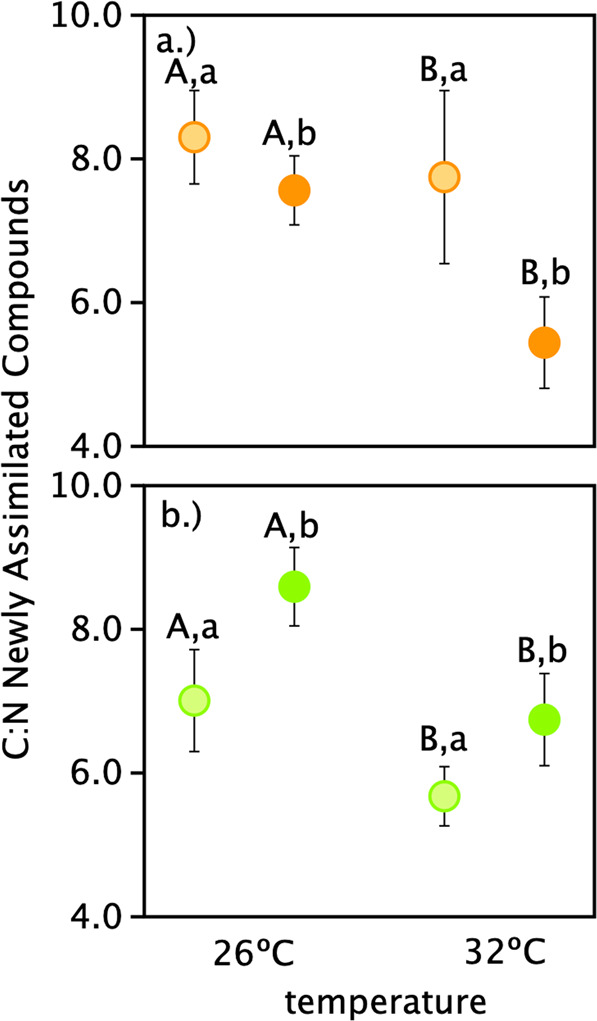
Stoichiometry of the response. Mean C:N (±95% confidence intervals) of newly assimilated compounds in *Cladocopium goreaui* (**a**; orange) and *Durusdinium trenchii* (**b**; green) incubated at 26 or 32 °C in the absence (light shades) or presence (dark shades) of interspecific competition. Letters indicate significant main effects of temperature and competition as determined by a two-factor ANOVA (*p* < 0.05).

Overall, the changes in nutrient assimilation significantly affected the C:N ratio of newly assimilated compounds (Fig. 2). For *C. goreaui*, ANOVA revealed a significant effect of temperature (*F*_(1,28)_ = 24.0, *p* < 0.001) and competition (*F*_(1,28)_ = 15.9, *p* < 0.001), without a temperature × competition interaction (*p* = 0.07; Fig. 2a). For *D. trenchii* there was a significant effect of temperature (*F*_(1,30)_ = 22.4, *p* < 0.001) and competition (*F*_(1,30)_ = 6.1, *p* = 0.02), without a temperature × competition interaction (*p* = 0.58; Fig. 2b).

## Discussion

Microbes compete in one of two ways: passive, exploitative competition, wherein one species consumes a limiting resource, restricting the availability of that resource to a competitor; and active, interference competition, in which individuals damage one another via antimicrobial secretions or contact-dependent killing [24]. Differentiating the two requires a clear accounting of the response of each individual competing species such as that made possible by FISH-Flow-SIA. Furthermore, environmental factors, such as temperature, can influence performance and a species’ ability to acquire resources and thus modulate competitive responses [3, 25]. In this study, we demonstrated that interspecific competition drives changes in the rates of carbon and nitrogen assimilation exhibited by two homologous *Symbiodiniaceae* species (Fig. 1a–d), which led to significant changes in the stoichiometry of newly assimilated compounds (Fig. 2). Monocultures of *C. goreaui* and *D. trenchii* were used to establish species-specific differences in metabolism and their “baseline” response to increased temperature when grown in isolation (Fig. 1a, b). We then measured each species’ response to competition relative to those baselines. Specifically, *C. goreaui* exhibited traits of exploitative competition, increasing the proportion of nitrogen rich biomolecules—proteins and nucleic acids which are all essential for cell division [9], but only at increased temperature (Fig. 2a). Meanwhile, an increase in the relative proportion of newly assimilated carbon-rich biomolecules in *D. trenchii* when in the presence of *C. goreaui* is reflective of investments in carbohydrates and lipids for future growth ([9]; Fig. 2b). While herein limited to species in culture, these findings provide a foundation and a methodology for considering the role of competition in microbial community structure and succession *in hospite*.

### Functional variability

The distribution of species within their environment, whether free living or *in hospite*, will be affected by species traits and their functional variability. Both light and temperature are known to affect *Symbiodiniaceae* distributions at both the free living and *in hospite* stages (e.g., [12, 26]). At the 26 °C baseline, carbon assimilation for both *C. goreaui* and *D. trenchii* was similar but *D. trenchii* had a higher rate for nitrogen. Increased temperature further distinguished the metabolic strategy of the two species. *C. goreaui* increased both carbon and nitrogen assimilation at 32 °C, with a relatively small subsequent change in C:N (Fig. 2a). Documented effects of moderate temperature stress on *Symbiodiniaceae* include loss of photosynthetic membrane integrity (hours–days), buildup of reactive oxygen species (days), loss of photosynthetic function (days–weeks), and cell death (days–weeks) (reviewed in [27, 28]); each of these are associated with a decrease in nutrient assimilation. Thus, the punctuated increase in metabolic rate of *C. goreaui* in response to a temperature increase is unlikely driven by an impairment of photosynthesis, particularly within such a short (4 h) timeframe. At higher temperatures *D. trenchii* increased nitrogen, but not carbon, assimilation, resulting in a relatively large decrease in C:N of newly assimilated compounds (7.0 vs. 5.7; Fig. 2b), a response known to precede cell division [9, 29, 30]. The differences in baseline nutrient demand demonstrated here not only provide insight into how these species respond to temperature, but given oligotrophic nature of tropical marine habitats, this functinal variation in nutrient aquisition and assimilation can be another important axis along which niche partitioning may be taking place [31].

### Interspecific competition

In this study, we found species-specific metabolic responses to the presence of competition that manifested within just 4 h of exposure. For example, in the presence of *C. goreaui*, *D. trenchii* significantly increased carbon assimilation under both temperature treatments. This drove a proportional shift toward newly assimilated compounds that were higher in C:N, a well-known response by algae to nitrogen limitation and evidence of a shift toward investments in storage compounds that would be beneficial when environmental and/or ecological conditions became more favorable (e.g., when released from competition; [9, 32]). Contrastingly the competitive response of *C. goreaui* was to increase nitrogen assimilation by 30% and reduce carbon assimilation by 14%, but this response was temperature specific occurring only at 32 °C (Fig. 1a, b). Thus, despite the increase in baseline metabolic demand observed in *C. goreaui* monocultures under thermal stress, competing *C. goreaui* invested proportionally more into lower C:N compounds, which suggests a boosted investment in cell replication. Similar shifts to increased growth rates as a mechanism for resource exploitation have been observed in many microbial systems in competition [24]. However, given the culture media conditions, the short (4-h) timeframe of our study, and the mismatch in species sensitivity to temperature it is unlikely that *C. goreaui* could have been effective in exploiting nitrogen to the point of limitation for *D. trenchii*. Instead of resource exploitation/limitation, the competitive response of *D. trenchii* may be the result of interference competition. The co-option of signaling molecules (i.e., micro-RNAs) and/or potential allelopathic compounds, which is widespread among dinoflagellates [33, 34], could instead be implicated and deserve further attention. In this case, however, the ability of *D. trenchii* to exhibit enhanced metabolism (carbon assimilation) under competition without trade-offs in nitrogen assimilation may be the key to its ability to persist at background levels [35, 36]. Interestingly, *C. goreaui* is the dominant symbiont type in many corals at ambient temperatures [37] and was not sensitive to interspecific competition at 26 °C (Fig. 1c).

### Implications for symbiosis

As in every ecological system, competitive intensity and outcomes will be determined by environmental conditions. Thus, while experiments with symbionts in culture are an essential first step for understanding competitive potential among symbionts outside of host influence, the underlying mechanisms of competition both inside and outside of hosts require further investigation across various hosts and environmental conditions. The cultures used in this study were chosen for the fact that they can co-occur and transition in and out of dominance within a host [36]. At ambient temperatures (26–28 °C) corals that host predominantly *C. goreaui* receive more carbon [38] and nitrogen [39], and grow faster [40, 41], but are generally more sensitive to heat stress (30–32 °C) and bleaching relative to corals that host predominantly *D. trenchii* ([41, 42], but see [43]). The intrinsic differences between multiple species and how they respond to competition in culture can provide baseline expectations of how functional and ecological traits contribute to the ultimate composition of symbiont communities *in hospite*. For example, in N-limited coral reef habitats, symbionts with low nitrogen demand will have a relatively lower sensitivity to nutrient limitation and therefore higher competitive potential in oligotrophic environments [42]. Nitrogen limitation within coral cells has recently been confirmed by NanoSIMS with density dependent effects on symbiont populations [8]. The lower baseline requirement for nutrients observed in *C. goreaui* relative to *D. trenchii* at 26 °C correlates with their tendency to dominate coral hosts under ambient temperature conditions [19]. In response to thermal stress, increased respiration in *Cladocopium* species led to a shift toward parasitism in its relationship with host *Orbicella faveolata* [30] demonstrating that imbalances in holobiont nutrient recycling contribute to the destabilization of a healthy symbiotic state. In culture, the metabolic response of *C. goreaui* was especially pronounced where both temperature stress and competition were present which would further divert resources from the symbiosis. However, understanding how competitive traits translate to nutrient sharing in symbiosis will require the application of FISH-Flow-SIA to *in hospite* systems with simultaneous measures of host tissue enrichments at macro scales.

*D. trenchii*, confers increased temperature tolerance to some *Acropora spp.* and other coral hosts globally. Based on these patterns observed *in hospite*, we expected *D. trenchii* to be relatively insensitive to increased temperature [39] with a temperature-dependent response to competition. Instead, *D. trenchii*, assimilated more nitrogen at 32 °C and  was consistently sensitive to competition across temperatures (Fig. 1c, d). Thus, rather than outcompeting *C. goreaui* in any scenario, it is more likely that *D. trenchii* is the “last man standing” as it is able to tolerate and then repopulate coral tissues under conditions unsuitable for other symbionts. In other words, this species seems well suited to proliferate only in the absence of other strains, e.g., under extreme environmental conditions [35], following bleaching [18], and in newly available host habitat [44]. Indeed, during the initial colonization of coral recruits *D. trenchii* often remained at lower than expected densities when other symbionts were present despite being able to fully colonize corals in their absence [44]. When temperature stress is removed, *D. trenchii* is driven to background levels by competitive dominance of other species, which replace *D. trenchii* over time [18]. Relative to *C. goreaui*, *D. trenchii* showed higher baseline nitrogen assimilation at both temperatures which aligns with the increased cost and potential consequences demonstrated for corals of associating with thermally tolerant symbionts [38, 39].

Evidence of symbiont competition for host habitat [44] and resources [8] has been accumulating with potential negative effects on the coral host. The competitively driven changes in symbiont metabolism revealed herein would have downstream effects on the coral symbiosis potentially through both resource diversion, allelopathy, and/or competitive outcomes. Furthermore, variation in functional traits, and the pattern of dominance of *C. goreaui* in corals at ambient temperatures and *D. trenchii* in corals subject to thermal stress, support the potential for competition to play a role in structuring *in hospite* communities. In the face of climate change, interventions to accelerate the adaptation of corals to withstand stress are warranted [45], with functional probiotics as a promising tool [46, 47]. However, if a microbial partner is not competitive across a range of ambient conditions, it will only occasionally achieve its desired function [48]. This presents a major obstacle to the efficacy of assisted evolution of mutualistic and stress resistant *Symbiodiniaceae*.

There is no shortage of interesting ecological and evolutionary questions that have arisen in parallel with the discovery of broad genetic diversity within microbial communities. However, isolating the function of specific microbes within a complex microbiome is challenging [21, 49]. We have demonstrated that established methodologies (FISH labeling, flow cytometry, and SIA) applied in a novel manner provides a new perspective into how competition among microbial symbionts may impact trajectories of holobiont health. The limitations of this technology to target a single species at a time are driven by the high autofluorescence across a broad spectrum [50] will be overcome as fluorescent labeling technologies improve. The application of FISH-Flow-SIA to other taxonomic groups of microbes in corals and other hosts/environments requires only the redesign and validation of probe sequences for any target. Furthermore, probe design within FISH-Flow-SIA allows for flexibility in the taxonomic resolution of microbial targets and the ability to group taxa at higher or lower taxonomic levels. We believe that these methods and complementary emerging technologies (e.g., NanoSIMS combined with FISH labeling) will underpin a more comprehensive understanding of symbioses across systems, which includes revisiting not only the role of interspecific competition, but also a hosts’ ability to monopolize competitive outcomes through nutrient regulation.

## Supplementary information

Supplementary Materials

## Data Availability

Raw data are available on GitHub repository at github.com/shelby26/FFS.
