# Supplementary material for: Competitive traits of coral symbionts may alter the structure and function of the microbiome
Source: ISME J. 2020 Jun 9;14(10):2424–32. doi: 10.1038/s41396-020-0697-0 (PMC7490369; doi:10.1038/s41396-020-0697-0)
Supplement: Supplementary file 1 — Supplementary Materials [file 41396_2020_697_MOESM1_ESM.docx]

SUPPLEMENTARY MATERIALS

Title: Competitive traits of coral symbionts may alter the structure and function of the microbiome.

Appendix 1. Methodological Results

Efficacy of FISH-Flow-SIA

*FISH labelling*

The breadth of autofluorescence in Symbiodiniaceae species at both green (green fluorescent proteins) and red (chlorophyll) wavelength traditionally requires depigmentation for FISH labelling which interferes with downstream analyses of stable isotopes. However, a natural decrease in autofluorescence at 550-600 nm allowed for the clear detection of probes modified with Alexa 546 and interrogated by a 561 nm yellow-green laser and emission screening with a 582/15 nm filter. This narrow range of low autofluorescence, however, limited our ability for multiplexing (distinguishing multiple species simultaneously). Because of these limitations, prior to probe hybridization, our co-culture samples were split in half; one half was labelled with the SymC-Alexa546 and the other with SymD-Alexa546 probe (for sequences and specificity data, see McIlroy et al. 2014). The monocultures were labelled with SymC-Alexa546 and SymD-Alexa546, where appropriate.

*Flow Cytometry*

Gating based on light scattering parameters (Forward Scatter - FSC and Side Scatter – SSC; Fig. S1a) allowed us to first screen cells for size and shape and to avoid the few cases of doublets, where multiple cells were stuck together (Fig. S1b, c). FISH-labelling efficiency was high (SymC = 96.5%, SymD = 88.2% in pure cultures) with probe-positive and probe-negative cells in co-culture clearly distinguished with an emission filter of 582/15 nm (Fig S1d, e). We targeted only probe-positive cells for sorting to ensure sample purity. Of the estimated 4 million cells added in each tracer pulse incubation, we were able to sort an average of 3.92 (s.d. = 0.99) and 3.75 (s.d. = 1.32) million probe positive cells per treatment for the *C. goreaui* monoculture (hybridized with SymC) and *D. trenchii* monoculture (hybridized with SymC), respectively. In the co-culture treatment, we sorted an average of 1.20 (s.d. = 0.20) and 0.83 (s.d.= 0.28) million probe positive cells from the SymC and SymD probe hybridizations, respectively (Fig. S2).

*SIA*

We evaluated the δ^13^C and δ^15^N on samples using a stable isotope ratio mass spectrometer. Following oven drying of sorted cells, sample weights were low (0.11 mg ± 0.11); low sample mass and the low relative mass of nitrogen within cells limits δ^15^N determination. Addition of a nitrogen carrier (0.18 mg of Na^14^NO_3_) was used to bring those values into appropriate ranges for EA-IRMS. We converted data to Atom Percent (AP) ^13^C and ^15^N, and then to calculated and removed the influence of the nitrogen carrier from sample values. For each monoculture SIA values for both carbon and nitrogen were not different for samples which had and had not undergone FISH-Flow-SIA, showing that the methodology did not interfere with isotope evaluations (Fig. S3). Furthermore, we emphasize the utility of comparing FISH-Flow sorted samples from bulk SIA analyses by showing that linear additive models are insufficient for estimating species specific contributions (Fig. S4).


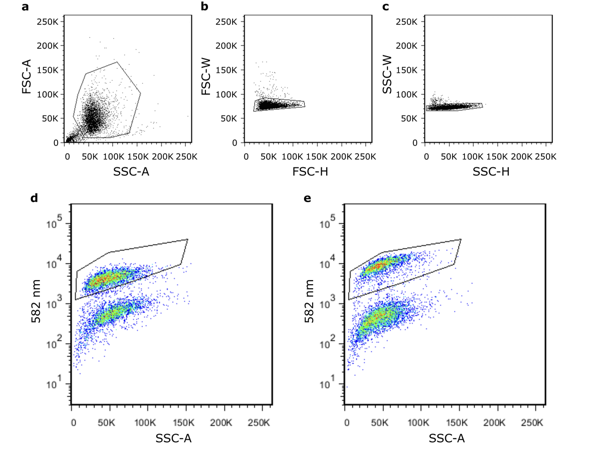


Fig. S1. Flow cytometry population heirarchy for co-culture sorting. (a) First gate isolates cells of interest based on size (forward scatter; FSC-A) and shape (side scatter; SSC-A), (b,c) A second forward scatter width (FSC-W) vs height (FSC-H) and third  gate side scatter width (SSC-W) vs height (SSC-H), screens out doublets, in which multiple cells are analyzed within a single drop. (d) Increased fluorescence at 582 nm fluorescence was used to distinguish cells from co-cultures labeled with either (d) SymC or (e) SymD.


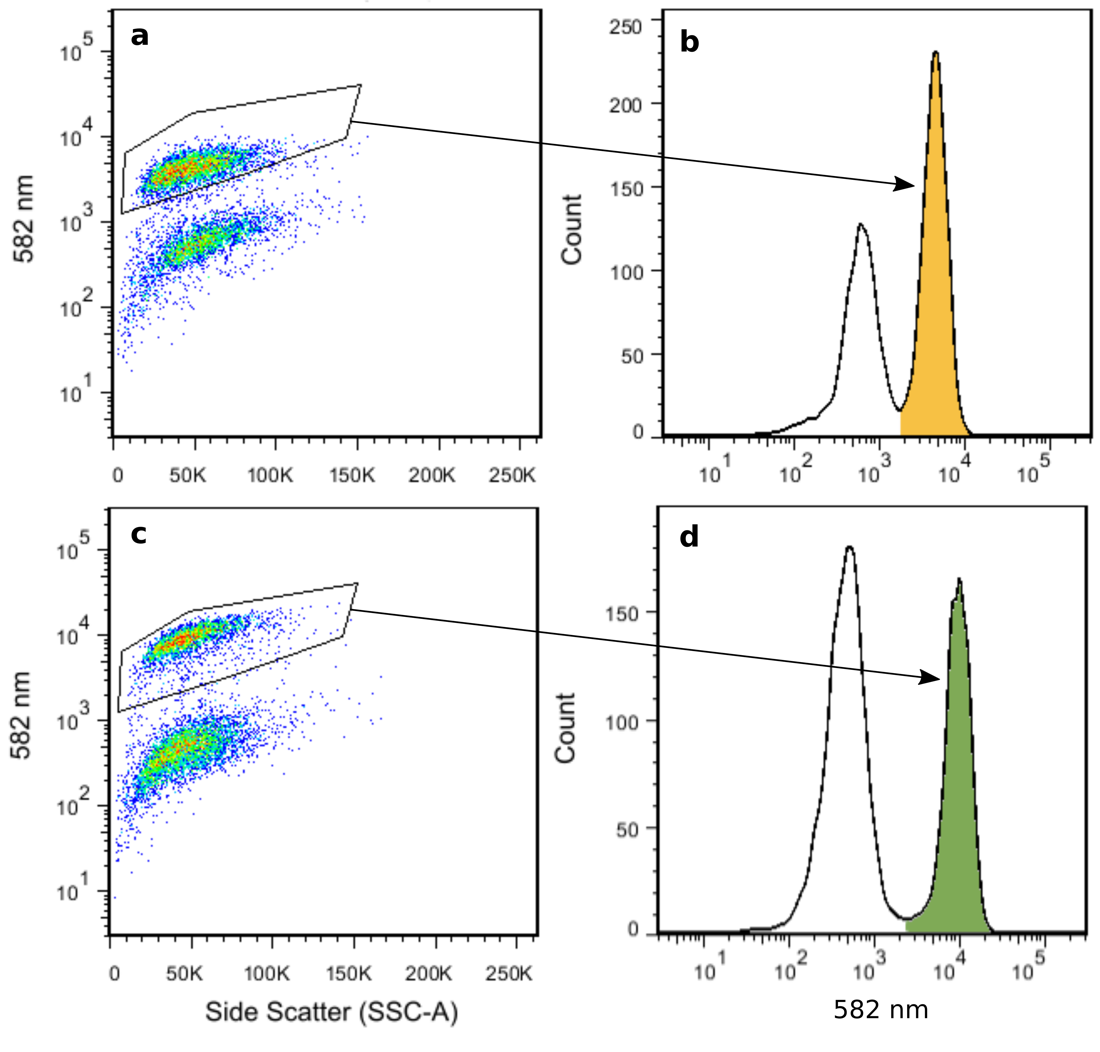


Fig. S2. The proportion of cells in a co-culture. Co-culture cells were labelled with *Cladocopium* specific probe, SymC (a & b) or *Durusdinium* specific probe, SymD (c & d). The figures on the left are FASC plots corrected for probe efficiency (96.5% for SymC and 88.2% for SymD). The figures on the right are the cell counts at 582 nm fluorescence, and the coloured area under the curve represents the number of cells labelled by (b) SymC (yellow) and (d) SymD (green). 57% of the co-culture were *C. goreaui* and 43% were *D. trenchii*, independent of the probe in used.


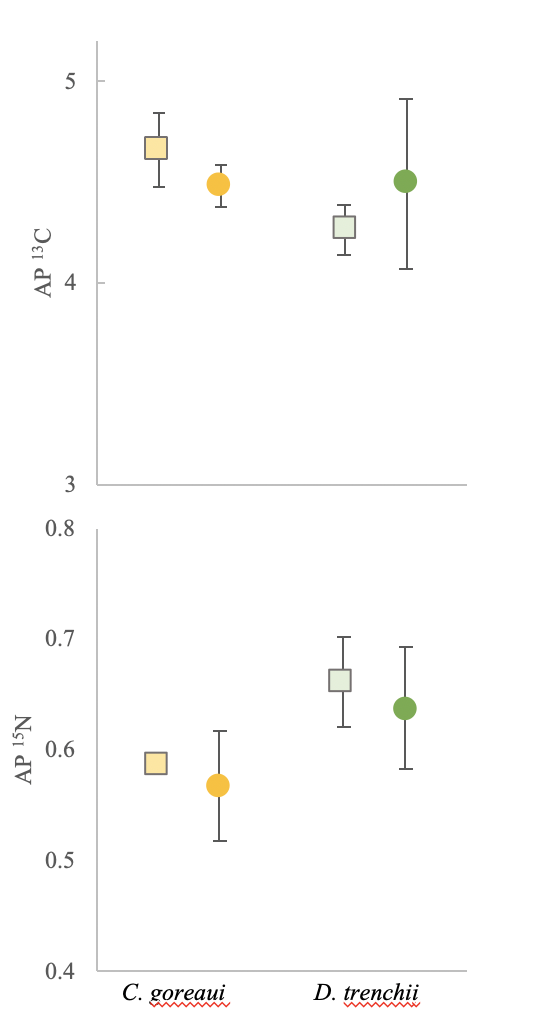


Fig. S3. Effect of FISH-Flow on SIA. Atom percent enrichment of cultures grown in batch culture at 26°C pulsed with isotopically enriched media. Values are shown for samples analyzed prior to (squares) and following (circles) FISH-Flow-SIA methodology. Error bars represent 95% confidence intervals.


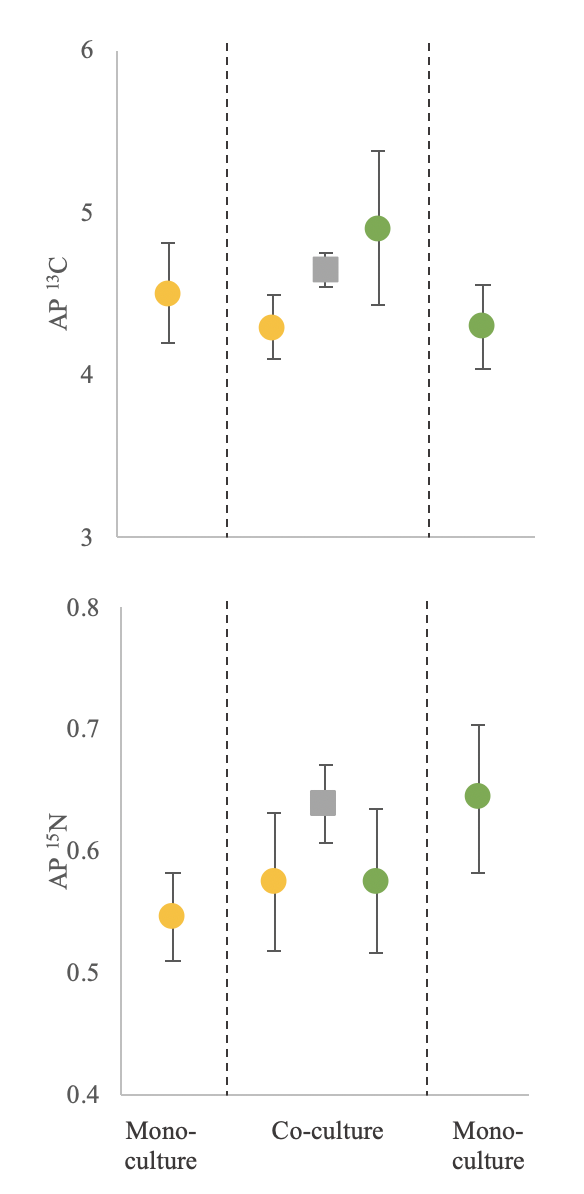


Fig. S4. Cryptic competition. Atom percent enrichment of cultures pulsed with isotopically enriched media at 26°C. Treatments included growth of *C. goreaui* (yellow) and *D. trenchii* (green) in monoculture and co-culture. Samples were processed with FISH-Flow-SIA methodology (circles) to isolate cells of interest (*C. goreaui* or *D. trenchii*), to compare with a non-processed bulk sample (grey square). Error bars represent 95% confidence intervals.


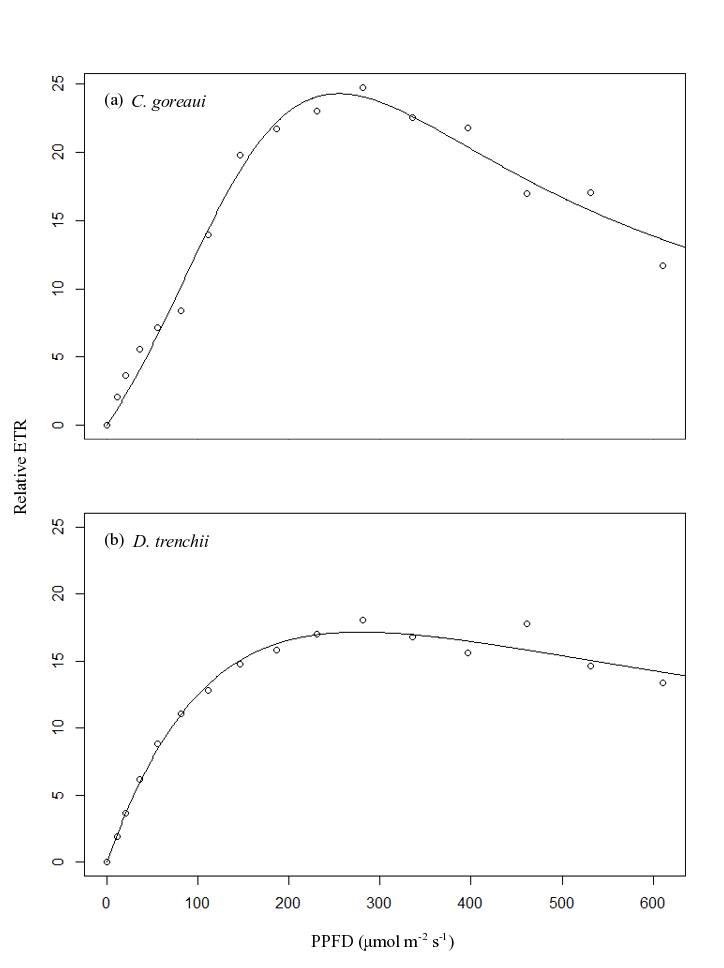


**Fig. S5. Light Acclimation.** Rapid light curves were generated with an imaging-PAM for a) *C. goreaui* and b) *D. trenchii.* The data was fitted with R package “phytotools” using the model of Eilers and Peeters 1988.

**Table S1. Providence of Symbiodiniaceae.** Cultures used in this experiment were obtained from the Symbiont Culture Facility at the Australian Institute of Marine Science in December 2016.

| Species | *Cladocopium goreaui* | *Durusdinium trenchii* |
| --- | --- | --- |
| Culture | SCF055 (clade C1) | SCF088 (clade D1a) |
| Host Species | *Acropora tenuis** | *Acropora muricata* |
| Location of Coral from which it was collected | Magnetic Island | Magnetic Island |

* collected under permit: *G10-33440.1*
